# Hexa-Histidine, a Peptide with Versatile Applications in the Study of Amyloid-β(1–42) Molecular Mechanisms of Action

**DOI:** 10.3390/molecules28207138

**Published:** 2023-10-17

**Authors:** Jairo Salazar, Alejandro K. Samhan-Arias, Carlos Gutierrez-Merino

**Affiliations:** 1 Departamento de Química, Universidad Nacional Autónoma de Nicaragua-León, León 21000, Nicaragua; 2 Departamento de Bioquímica, Universidad Autónoma de Madrid (UAM), C\Arzobispo Morcillo 4, 28029 Madrid, Spain; alejandro.samhan@uam.es; 3 Instituto de Investigaciones Biomédicas ‘Alberto Sols’ (CSIC-UAM), C\Arturo Duperier 4, 28029 Madrid, Spain; 4 Instituto de Biomarcadores de Patologías Moleculares, Universidad de Extremadura, 06006 Badajoz, Spain

**Keywords:** amyloid β(1–42), amyloid β(25–35), hexa-histidine, calmodulin, calbindin-D28k, hexa-histidine-tag, recombinant cytochrome *b*_5_ reductase, fluorescence, fluorescence resonance energy transfer

## Abstract

Amyloid β (Aβ) oligomers are the most neurotoxic forms of Aβ, and Aβ(1–42) is the prevalent Aβ peptide found in the amyloid plaques of Alzheimer’s disease patients. Aβ(25–35) is the shortest peptide that retains the toxicity of Aβ(1–42). Aβ oligomers bind to calmodulin (CaM) and calbindin-D28k with dissociation constants in the nanomolar Aβ(1–42) concentration range. Aβ and histidine-rich proteins have a high affinity for transition metal ions Cu^2+^, Fe^3+^ and Zn^2+^. In this work, we show that the fluorescence of Aβ(1–42) HiLyte^TM^-Fluor555 can be used to monitor hexa-histidine peptide (His_6_) interaction with Aβ(1–42). The formation of His_6_/Aβ(1–42) complexes is also supported by docking results yielded by the MDockPeP Server. Also, we found that micromolar concentrations of His_6_ block the increase in the fluorescence of Aβ(1–42) HiLyte^TM^-Fluor555 produced by its interaction with the proteins CaM and calbindin-D28k. In addition, we found that the His_6_-tag provides a high-affinity site for the binding of Aβ(1–42) and Aβ(25–35) peptides to the human recombinant cytochrome *b*_5_ reductase, and sensitizes this enzyme to inhibition by these peptides. In conclusion, our results suggest that a His_6_-tag could provide a valuable new tool to experimentally direct the action of neurotoxic Aβ peptides toward selected cellular targets.

## 1. Introduction

Amyloid β (Aβ) peptides are a hallmark of Alzheimer’s disease (AD). Aβ(1–42) is the prevalent Aβ peptide found in the amyloid plaques of AD patients [1]. Owing to the high affinity of Aβ(1–42) for Cu^2+^, Fe^3+^ and Zn^2+^, these metal ions are accumulated in Aβ plaques [2]. Despite the fact that Aβ plaques are cytotoxic, it has been proposed that Aβ plaques could serve as reservoirs to assemble small Aβ oligomers [3]. Aβ oligomers are the main neurotoxic forms of Aβ and have been linked to AD pathogenesis [4,5,6,7,8,9,10,11]. Furthermore, it has been shown that intraneuronal Aβ accumulation precedes the appearance of amyloid plaques or tangles in transgenic mice models of AD [6,12,13,14]. Aβ(25–35) is the shortest peptide that retains the toxicity of the full-length Aβ(1–42) peptide [15], and it has been suggested to be the biologically active region of Aβ(1–42) [16,17].

Aβ peptides bind to ganglioside-clustered raft-like membrane microdomains, which foster the formation of Aβ oligomers and fibrils in a cholesterol-dependent manner [18,19,20,21,22]. Indeed, dimeric nonfibrillar Aβ has been shown to accumulate in lipid rafts in the Tg2576 mouse model of AD [23], and exogenous oligomeric Aβ applied to neurons in culture concentrates in lipid rafts [24]. Moreover, plasma membrane lipid rafts play an active role in extracellular Aβ uptake and internalization in neurons [25]. After the uptake of extracellular Aβ(1–42) into neurons, the intracellular aggregates elicit neuronal damage and neurotoxicity [8,9,26,27]. Intracellular targets of Aβ(1–42) oligomers have been identified in the cytosol and subcellular organelles like the endoplasmic reticulum (ER) and mitochondria [28,29,30]. Of particular relevance are the intracellular targets of Aβ(1–42) oligomers that bind nanomolar concentrations of Aβ peptides, because concentrations of non-fibrillar Aβ peptides within the nanomolar range have been reported in the brain [31,32,33], and the critical concentration that induces Aβ(1–42) fibrillization lies in the submicromolar range [34,35]. Until now, dissociation constants lower than 10 nM have been reported for the complexes between Aβ peptides and a few intracellular proteins expressed in brain neurons, namely calmodulin (CaM) [36], cellular prion protein [37], glycogen synthase kinase 3α [38], tau [39], calbindin-D28k [40] and STIM1 [30]. However, in neurons, the concentration of CaM is in the micromolar range, which is orders of magnitude higher than the concentration of the other competing proteins in neurons listed above, with the exception of calbindin-D28k. Nevertheless, it is to be noted that, although calbindin-D28k is abundant throughout the central nervous system, including pyramidal hippocampal neurons and cortical neurons [41], the dissociation constant of the Aβ(1–42)/calbindin-D28k complex is around 10-fold higher than that of the Aβ(1–42)/CaM complex [40]. Due to this, CaM binds a large fraction of intracellular Aβ(1–42) in the nanomolar concentration range [27].

As CaM binds Aβ(1–42) and Aβ(25–35) peptides with very high affinity, i.e., with a dissociation constant ≈1 nM [36]. In a previous work, we used CaM as a template protein for the design of an antagonist peptide of Aβ(1–42) using docking approaches [40]. Fluorescence assays with Aβ(1–42) HiLyte^TM^-Fluor555 confirmed that this peptide efficiently inhibits the interaction between Aβ(1–42) and CaM [40]. Interestingly, the peptide designed to bind the 25–35 amino acid residues of Aβ(1–42) was experimentally found to also be a potent inhibitor of the interaction between Aβ(1–42) and calbindin-D28k [40], pointing out that the same amino acid domain of Aβ(1–42) is involved in its complexation with both CaM and calbindin-D28k. This bears a special relevance in AD, since these proteins play important roles in neuronal calcium signaling and the dysregulation of intracellular calcium homeostasis, and alterations of neuronal excitability have been shown to mediate Aβ neurotoxicity [42,43,44,45]. On the other hand, it is known that several endogenous neuropeptides can antagonize the actions of Aβ, both in animal models [46,47,48] and in cell cultures [49,50]. Therefore, the search for novel peptides that binds Aβ with high affinity can shed light on the research of endogenous neuropeptides with neuroprotection actions against Aβ. Histidine-rich proteins have a high affinity for Cu^2+^, Fe^3+^ and Zn^2+^ [51,52,53], like Aβ(1–42) [2,54]. Thus, polyhistidine motifs or peptides are good candidates as Aβ(1–42) antagonists and putative novel therapeutic agents in AD. Moreover, it has been noted that the hexa-histidine binding by monoclonal antibody C706, which recognizes the human Aβ peptide, mimics Aβ recognition [55].

In this work, we report that the fluorescence of Aβ(1–42) HiLyte^TM^-Fluor555 (dye covalently bound to ASP1 of Aβ) monitors hexa-histidine peptide (His_6_) interaction with Aβ(1–42) and the inhibition of the interaction between Aβ(1–42) and the proteins CaM and calbindin-D28k. Also, we found that the presence of a C-terminal His_6_-tag in human recombinant cytochrome *b*_5_ reductase (Cb5R) sensitizes its enzymatic activity to modulation by submicromolar Aβ(1–42) concentrations. In this work, we have used human recombinant Cb5R because the cytochrome *b*_5_ (Cb_5_)/Cb5R system has a relevant role in lipid metabolism, which is significantly altered in AD [56,57].

## 2. Results

### 2.1. Hexa-Histidine (His_6_) Interacts with Aβ(1–42) HiLyte^TM^-Fluor555

**Figure 1:**
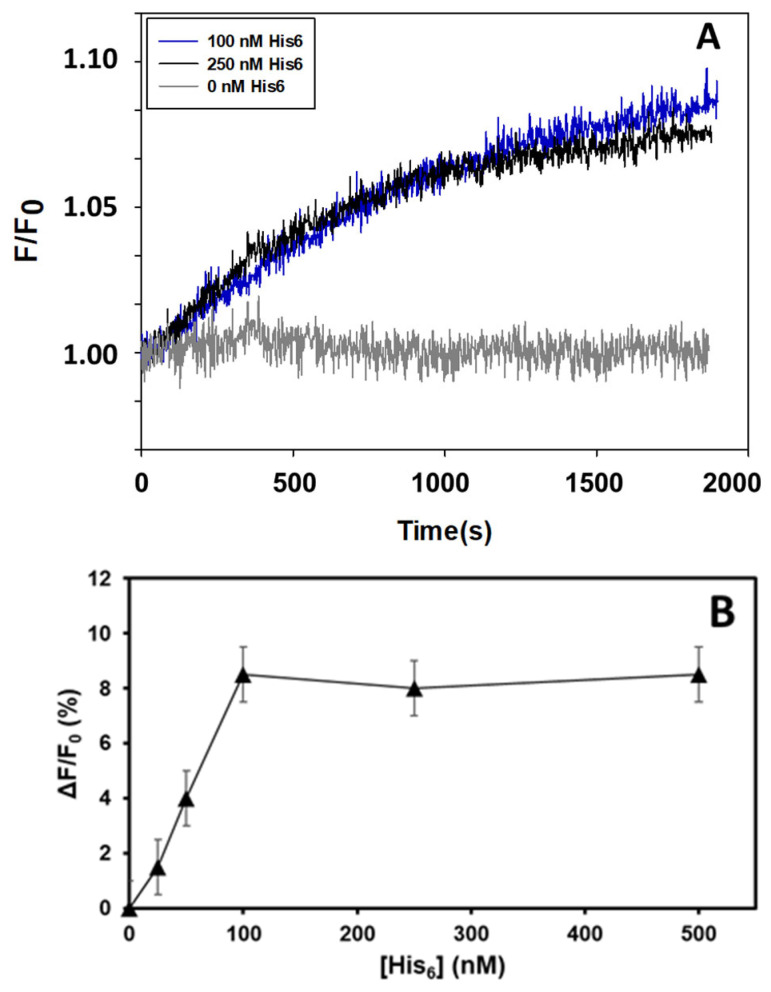
The fluorescence of Aβ(1–42) HiLyte^TM^-Fluor555 monitors its interaction with His_6_. (**A**) Kinetics of the increase in Aβ(1–42) HiLyte^TM^-Fluor555 fluorescence induced by the addition of His_6_. For a more direct evaluation of the magnitude of the increase in the fluorescence, the results are presented as the ratio between the fluorescence intensity at different times (F) and the fluorescence intensity at time 0 (F_0_). The kinetic traces shown are the means of triplicate experiments performed with 10 nM Aβ(1–42) HiLyte^TM^-Fluor555 and the concentrations of His_6_ indicated in the figure, which was added at time 0. The kinetic trace in gray color is the control with the addition of only the buffer in which His_6_ is prepared (0 His_6_). (**B**) Dependence upon the His_6_ concentration of the increase in the fluorescence intensity of Aβ(1–42) HiLyte^TM^-Fluor555 at the end of the kinetic of increase in fluorescence. Other experimental conditions used for these fluorescence measurements are given in Section 4.

The addition of submicromolar concentrations of His_6_ to a solution of 10 nM Aβ(1–42) HiLyte^TM^-Fluor555 increases the fluorescence intensity of the labelled peptide (Figure 1A). The kinetics of the increase in fluorescence, which monitors the interaction between His_6_ and Aβ(1–42) HiLyte^TM^-Fluor555, has an initial slower phase of 1–2 min and reaches a maximum of 8 ± 2% with a half-time of approximately 10 min. Although the kinetics are slightly faster with 0.25 μM of His_6_, the large overlap between the kinetic traces obtained with 0.1 and 0.25 μM of His_6_ points out that at the latter concentration, no new complexes are being formed. The results of titration with His_6_ shown in Figure 1B confirm the saturation of the maximum fluorescence change at a concentration of 0.1 μM of His_6_. The small value of the maximum fluorescence change indicates that His_6_ interaction with Aβ(1–42) elicits only a small change in the microenvironment of the HiLyte^TM^-Fluor555 dye, which is bound to ASP1 of Aβ(1–42). The short initial lag phase suggests that conformational changes in His_6_ and/or Aβ(1–42) HiLyte^TM^-Fluor555 are needed for a better coupling between both molecules. The dependence upon the His_6_ concentration of the increase in fluorescence intensity at 2000 s of the kinetic of the increase in fluorescence is shown in Figure 1B. Taking into account that the noise of the fluorescence signal introduces an error close to ±2% in the differences between fluorescence intensity readings, the 50% of the maximum fluorescence change is reached with a concentration of ≈50–60 nM of His_6_, i.e., the saturation of the fluorescence change is observed at ratios ≥10 molecules of His_6_ per Aβ(1–42) HiLyte^TM^-Fluor555 monomer.

The experimental results shown above prompted us to perform docking between His_6_ and/or Aβ(1–42). The analysis of docking has been performed using the MDockPeP Server, as indicated in Section 4. The structure of Aβ(1–42) registered with PDB ID: 1IYT in UniPro Databank has been selected for this docking work due to the following reasons: (1) at the concentration of 10 nM used in these experiments, the Aβ(1–42) peptide must be largely in the monomeric state because the dissociation constant for Aβ(1–42) dimers is similar to that reported for Aβ(1–40) dimers, i.e., around 198 ± 43 nM [58], and (2) we found in a previous work that this structure of the Aβ(1–42) monomer is a good template structure for the design of an efficient peptide antagonist for the interaction between Aβ(1–42) and CaM [40]. The results generated 10 possible structural models for the complex formation, which can be grouped into three clusters, as shown in Table 1 and Supplementary Tables.

**Figure 2:**
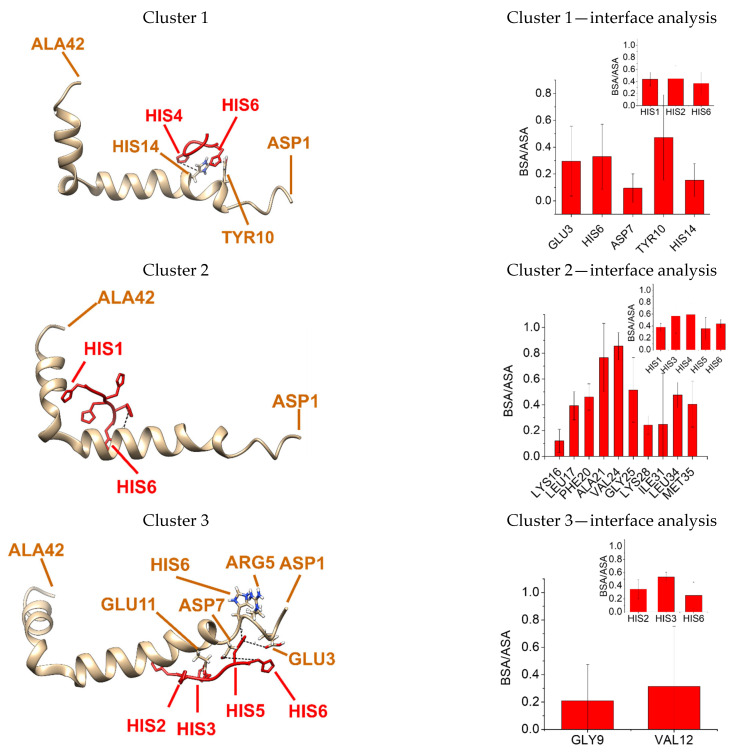
Clusters of the structures generated using the MDockPeP Server for the complex between 1His_6_ and the structure of Aβ(1–42) registered with PDB ID: 1IYT in UniPro Databank analyzed with PDBePISA. Aβ(1–42) backbone is shown in brown and 1His_6_ peptide is shown in red for the representative models with the lowest estimated binding energy of cluster1, cluster 2 and cluster 3, model 7, model 9 and model 3, respectively. H-bonds between amino acids of both structures are shown as a dotted black line.

In cluster 1, His_6_ interacts with the Aβ(1–42) domain close to the NH_2_-terminus and involves the short α-helix and the part of the long α-helix near the structural tilt of this peptide. Cluster 1 gathers models 1, 4, 7 and 8 and presents an estimated free energy for the complex formation of −6.8 kcal/mol, which is that found for the lowest energy model of the cluster (model 7) (Figure 2, cluster 1). The interacting interface in cluster 1 comprises the following amino acid residues of the Aβ peptide: LYS16, LEU1, PHE20, ALA21, VAL24, GLY25, LYS2, ILE31, LEU34 and MET35 and HIS1, HIS3, HIS4, HIS5 and HIS6 residues of the His_6_ peptide. Buried surface area/accessible solvent area ratio allowed us to rank those amino acid residues with higher participation in the interaction with Aβ peptide: VAL24 > ALA21 > GLY25 > LEU34 > PHE20 > MET35 > LEU17 > ILE31 > LYS28 > LYS16 and a similar participation for the HIS1, HIS3, HIS4, HIS5 and HIS6 of the His_6_ peptide. Several H-bonds and salt bridges were found in models from cluster 1 but without sharing the same amino acid residues among themselves (Supplementary Table S1).

Cluster 2 is the second most probable cluster and is representative of another four of the ten most probable outcomes generated using the MDockPeP Server. In cluster 2, His_6_ interacts with the Aβ(1–42) domain close to the COOH-terminus. Cluster 2 gathers models 2, 5, 9 and 10. Analysis with PDBePisa estimated free energy for the complex formation of −4.8 kcal/mol, which is that found for the lowest energy model of the cluster (model 9) (Figure 2, cluster 2). The interacting interface in cluster 2 comprises the following amino acid residues of the Aβ peptide: GLU3, HIS6, ASP7, TYR10, HIS14 and HIS1, HIS2, HIS6 residues of the His_6_ peptide. Buried surface area/accessible solvent area ratio allowed us to rank those amino acid residues with higher participation in the interaction with Aβ peptide: TYR10 > HIS6 > GLU3 > HIS14 > ASP7 and similar participation for the HIS1, HIS2 and HIS6 of the His_6_ peptide. Several H-bonds and salt bridges were also found in models from cluster 2, but as also accounting for cluster 1, they did not share similarities among themselves (Supplementary Table S2). A third cluster was found based on models sharing similarities in their interaction. Cluster 3 is representative of two of the ten most probable structures generated using the MDockPeP Server and predicts that His_6_ may also interact with the long α-helix part closer to the COOH-terminus of Aβ(1–42). Cluster 3 gathers models 3 and 6. Analysis with PDBePisa estimated free energy for the complex formation of −2.6 kcal/mol, which is that found for the lowest energy model of the cluster (model 3) (Figure 2, cluster 3). The interacting interface in cluster 3 comprises the GLY9 and VAL184 amino acid residues of Aβ peptide and HIS1, HIS2, HIS6 residues of the His_6_ peptide. Buried surface area/ accessible solvent area ratio indicates that both amino acid residues of Aβ peptide (GLY9 and VAL184) participate similarly in the interaction with the His_6_ peptide and Aβ peptide. Several H-bonds and salt bridges were also found in models from cluster 3 without sharing similarities among themselves (Supplementary Table S3).

We can summarize that the analysis in silico allows us to predict large conformational plasticity in the His_6_/Aβ(1–42) complex, suggesting that the predominant conformation of this complex could be strongly affected by its microenvironment. In addition, docking results raise the possibility that the Aβ(1–42) monomer can bind up to 2–3 molecules of His_6_.

### 2.2. His_6_ Antagonizes the Interaction of Aβ(1–42) HiLyte^TM^-Fluor555 with CaM and Calbindin D28k

In previous works, we have shown that Aβ(1–42) binds with high affinity to CaM and calbindin-D28k [36,40]. Since model 1 predicts a strong interaction of His_6_ with the domain of Aβ(1–42) more directly involved in its interaction with CaM and calbindin-D28k, in this work, we have experimentally assessed the possibility that His_6_ could antagonize the interaction of Aβ(1–42) with these proteins.

**Figure 3:**
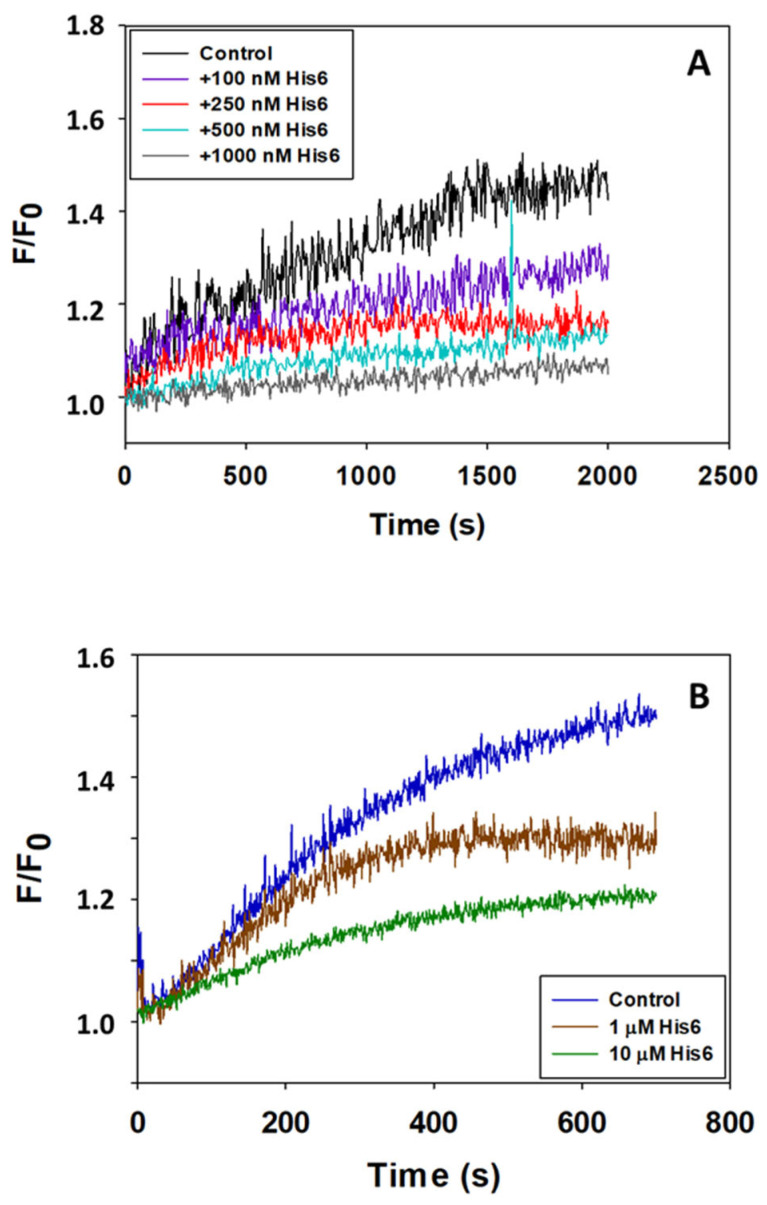
The peptide His_6_ antagonizes the interaction of Aβ(1–42) with calbindin-D28k and CaM. (**A**) Effect of increasing concentrations of His_6_ on the kinetics at an increase of 10 nM Aβ(1–42) HiLyte^TM^-Fluor555 fluorescence after adding 5 nM calbindin-D28k at time 0. (**B**) Effect of increasing concentrations of His_6_ on the kinetics at an increase of 10 nM Aβ(1–42) HiLyte^TM^-Fluor555 fluorescence after adding 5 nM CaM at time 0. The results are presented as the ratio between the fluorescence intensity at different times (F) and the fluorescence intensity at time 0 (F_0_), and the kinetic traces shown in this figure are the means of experiments performed in triplicate. The color code used for the assayed concentrations of His_6_ is given in the inset of the figures. Other experimental conditions used for these fluorescence measurements are given in Section 4.

The kinetics of the interaction of the fluorescence derivative of Aβ(1–42), Aβ(1–42) HiLyte^TM^-Fluor555, with CaM and calbindin-D28k can be monitored by the fluorescence intensity changes of the dye HiLyte^TM^-Fluor555 [40]. A preincubation of 15 min with His_6_ was included in the experimental design to prevent a distortion of the kinetics of fluorescence increase produced by CaM or calbindin-D28k by the change of fluorescence elicited by His_6_. Figure 3 shows that His_6_ attenuates the increase in fluorescence intensity Aβ(1–42) HiLyte^TM^-Fluor555 after adding either calbindin-D28k or CaM. This is an effect dependent upon the concentration of His_6_. As shown in Figure 3, the maximum change of the fluorescence intensity and the rate of the kinetic process are decreased by His_6_, albeit with different efficacy. These results yield His_6_ concentrations for a 50% attenuation of the maximum change of fluorescence intensity of ≈0.1 and 1 μM for the interaction of Aβ(1–42) with calbindin-D28k and CaM, respectively. Therefore, His_6_ behaves as an antagonist of the interaction of Aβ(1–42) with these proteins, although with different potency in the submicromolar-to-micromolar concentration range.

### 2.3. Submicromolar Concentrations of Aβ Peptides Inhibit the Reduction in Cb_5_ Catalyzed by Purified Recombinant His_6_-Tagged Cb5R, and No Inhibition Is Observed after Deletion of the His_6_-Tag

In order to evaluate the ability of His_6_ to sensitize proteins to Aβ peptides, we have used human recombinant Cb5R with a His_6_-tag covalently linked to the NH_2_-terminus amino acid, expressed and purified as described in Section 4. The NADH-dependent reduction in Cb_5_ has been measured, following protocols used in previous works, as indicated in Section 4.

**Figure 4:**
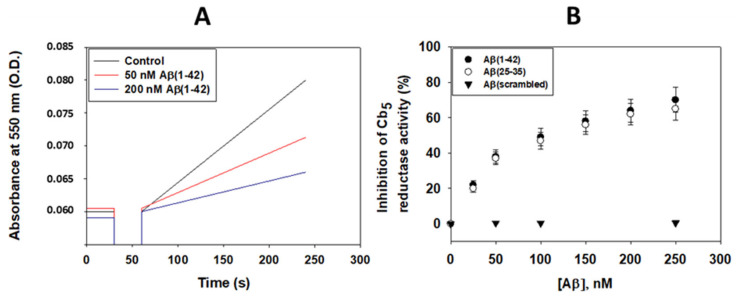
Aβ(1–42) and Aβ(25–35) inhibit the NADH-dependent Cb_5_ reductase activity of the human recombinant Cb5R with a His_6_-tag. (**A**) Kinetics of Cb_5_ reduction by the human recombinant Cb5R with a His_6_-tag in the absence and presence of 50 and 100 nM Aβ(1–42). (**B**) Dependence upon the concentration of Aβ(1–42), Aβ(25–35) and scrambled Aβ(1–42) of the inhibition of the Cb_5_ reductase activity of the human recombinant Cb5R with a His_6_-tag. The results shown are the means ± standard error of the mean (S.E.M.) of experiments performed in triplicate. Other experimental conditions used for these assays are given in the Section 4.7.

Representative kinetic traces of the rate of NADH-dependent reduction in Cb_5_ are presented in Figure 4A. A specific activity of 7.9 ± 0.7 µmoles of the reduced Cb_5_/min/mg of Cb5R at 25 °C has been calculated from initial rate measurements for the samples of human recombinant C-terminal His_6_-tagged Cb5R. Also, the kinetic traces shown in Figure 4A highlight the potent inhibition of this activity by nanomolar Aβ(1–42) concentrations. As up to 2 μM of Aβ(1–42), the highest concentration tested in this work, we have not detected any alteration of the absorbance spectrum of Cb_5_, the possibility that this inhibition could be due to an Aβ(1–42)-induced conformational change in the microenvironment of the heme group of Cb_5_ can be discarded. Moreover, the inhibition is seen from the first absorbance measurements after the addition of Aβ(1–42), implying its rapid binding to the inhibitory site in the Cb5R. Indeed, the observed inhibition was the same without or with up to 30 min preincubation of the Cb5R with 50 and 100 nM of Aβ(1–42). Next, we removed the His_6_-tag from human recombinant Cb5R as described in detail in Section 4. We found that the addition of up to 2 μM of Aβ(1–42) and of Aβ(25–35) does not produce a statistically significant inhibition of the rate of NADH-dependent reduction in Cb_5_ by human recombinant Cb5R minus a His_6_-tag, i.e., less than 10% inhibition. Of note, a 2 micromolar concentration of Aβ(1–42) oligomers is higher than those that can be expected intracellularly in AD, since the induction of fibrillization only requires submicromolar concentrations of Aβ(1–42) [34,35].

The results of the dependence upon the concentration of Aβ(1–42) and Aβ(25–35) of the inhibition of the NADH-dependent reduction in Cb_5_ by human recombinant Cb5R with a His_6_-tag are shown in Figure 4B. These results showed that Aβ(1–42) and Aβ(25–35) are equally potent as inhibitors of this activity, without statistically significant differences between both datasets. The inhibitory effect is specific of neurotoxic Aβ peptides, because a peptide with a random sequence of the 42 amino acids of Aβ (scrambled Aβ), which has been found non-toxic in previous works [27,30], does not inhibit this activity (Figure 4B). The results of inhibition by Aβ(1–42) and Aβ(25–35) fit well to the equation for one inhibitor binding site, with an inhibition constant (Ki) between 50 and 60 nM of Aβ monomers, and maximum inhibition (Imax) between 75 and 80%. Of note, this Ki value is nearly 10-fold higher than the Cb5R concentration used in these assays, and, therefore, the contribution of the peptide bound to Cb5R to the total Aβ concentration is lower than 10% at half of the saturation of the inhibition curve.

Combining these kinetic results, it merges the conclusion that the Cb_5_ reductase activity of the His_6_-tagged Cb5R is strongly sensitized to neurotoxic Aβ peptides.

### 2.4. The Binding of Aβ(1–42) and of Aβ(25–35) to the Human Recombinant Cb5R with a His_6_-tag Increases the Fluorescence of Its Prosthetic Group FAD

In previous works [59,60], we have shown that the fluorescence of the prosthetic group FAD can be used to monitor conformational changes of Cb5R, which impair its catalytic activity.

**Figure 5:**
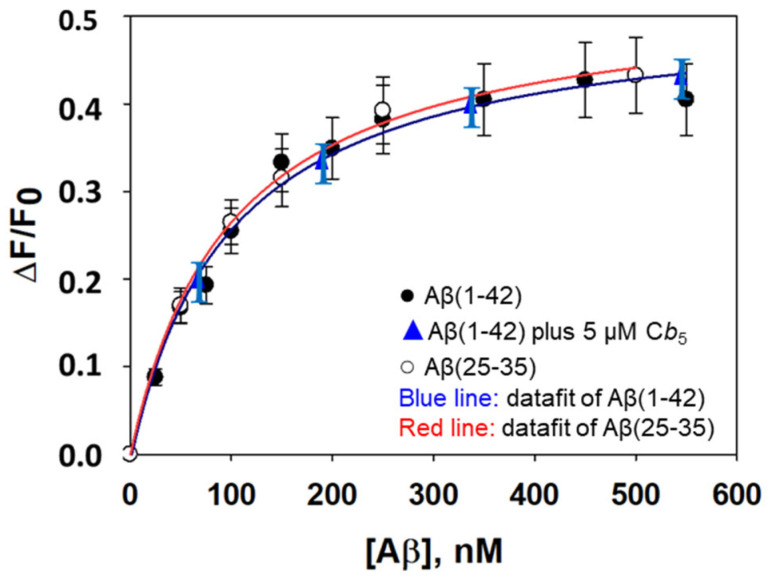
Dependence upon the concentration of Aβ(1–42) and Aβ(25–35) of the increase in FAD fluorescence (ΔF/F0) bound to the human recombinant Cb5R with a His_6_-tag. There was not a significant difference between the titration of FAD fluorescence with Aβ(1–42) in the absence (filled black circles) and the presence of 5 μM Cb_5_ (blue triangles). The lines are the results obtained via non-linear regression fit to the equation for 1:1 ligand binding site per protein molecule. The fit of the data yielded the following results: Y = −0.0095 + [0.5227 × x/(96.92 + x)] (R^2^ = 0.9870) for Aβ(1–42), and Y = −0.0019 + [0.5311 × x/(99.253 + x)] (R^2^ = 0.9974) for Aβ(25–35). The results shown are the means ± S.E.M. of experiments performed in triplicate. Other experimental conditions used for these fluorescence measurements are given in Section 4.

In this work, we found that the addition of Aβ(1–42) and of Aβ(25–35) produces an increase in the FAD fluorescence of human recombinant Cb5R with a His_6_-tag without a significant shift of the emission peak wavelength due to a conformational change that leads to an increase in the quantum yield of FAD bound to the Cb5R. Figure 5 shows that the FAD fluorescence increase depends on the concentration of the Aβ(1–42) and of Aβ(25–35) peptides. Moreover, both the magnitude and the dependence upon the Aβ peptide concentration of the change of FAD fluorescence are not significantly different between Aβ(1–42) and Aβ(25–35). Of note, this is an effect produced by the assayed neurotoxic peptides because up to 1 μM of “scrambled” Aβ(1–42) peptide does not elicit a statistically significant change in FAD fluorescence. The results obtained for the dependence of the increase in FAD fluorescence upon the concentration of Aβ(1–42) and of Aβ(25–35) fit well into the equation for one-site ligand–protein interaction (Figure 5). The non-linear fit of the results to this equation yields an increase of 41 ± 4% of the FAD fluorescence at saturation with these peptides, and an Aβ concentration for 50% of the effect of 75 ± 5 nM of peptide monomer. Since the concentration of Cb5R in the fluorescence assays is 32 ± 3 nM, the real free-peptide concentration at 50% saturation allows us to calculate an apparent dissociation constant (K_d,app_) of 59 ± 5 nM for the complex between the Cb5R and the Aβ peptide. This K_d,app_ value is within the range of values obtained for the Ki, pointing out that the binding of the Aβ peptide to this site causes the inhibition of the NADH-dependent Cb_5_ reductase activity of the human recombinant Cb5R with a His_6_-tag. Since the dependence of the FAD fluorescence upon the concentration of Aβ(1–42) was the same in the absence and presence of 5 μM of Cb_5_, the possibility that Aβ could interact with the Cb_5_ binding domain in the Cb5R can be excluded. Also, this result indicates that Aβ(1–42) does not bind to Cb_5_, resulting in further experimental support to our conclusion derived from the lack of effect of Aβ(1–42) on the absorbance spectrum of Cb_5_ (see above).

**Figure 6:**
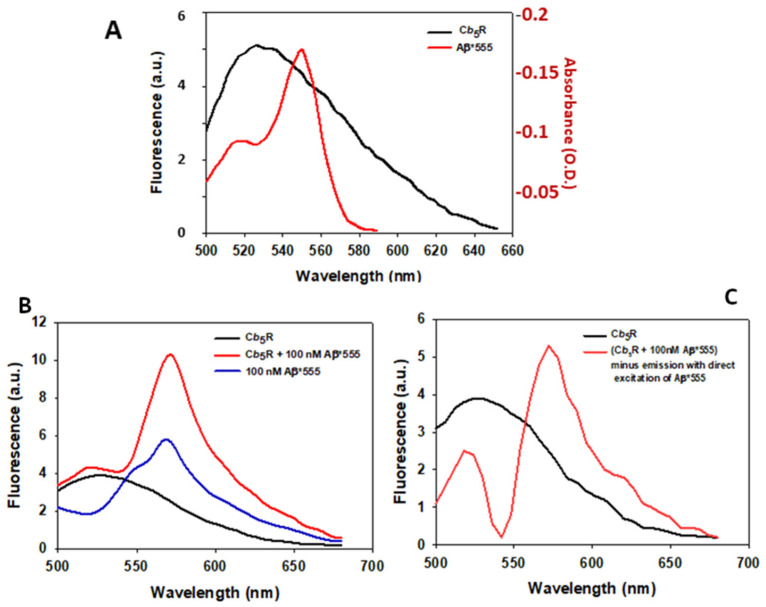
FRET between FAD bound to the human recombinant Cb5R with a His_6_-tag and Aβ(1–42) HiLyte^TM^-Fluor555. (**A**) Emission spectrum of the human recombinant Cb5R with a His_6_-tag with an excitation wavelength of 460 nm (black line) and absorption spectrum of Aβ(1–42) HiLyte^TM^-Fluor555 (Aβ*555, red line). (**B**) Emission spectra of the human recombinant Cb5R with a His_6_-tag in the absence (black line) and presence of 100 nM of Aβ(1–42) HiLyte^TM^-Fluor555 (Aβ*555) (red line), and of 100 nM Aβ(1–42) HiLyte^TM^-Fluor555 (Aβ*555) in the absence of Cb5R (direct excitation spectrum, blue line). Excitation wavelength = 460 nm, excitation and emission slits =10 nm. (**C**) Emission spectrum of the human recombinant Cb5R with a His_6_-tag (black line) and the result of the emission spectrum of (Cb5R plus 100 nM of Aβ(1–42) HiLyte^TM^-Fluor555) after the subtraction of the emission spectrum of 100 nM Aβ(1–42) HiLyte^TM^- Fluor555 in the absence of Cb5R (red line). Other experimental conditions used for these fluorescence measurements are given in Section 4.

In order to further assess the interaction between Aβ(1–42) and human recombinant Cb5R with a His_6_-tag, we have used the fluorescent derivative Aβ(1–42) HiLyte^TM^-Fluor555. This compound can act as a fluorescence resonance energy transfer (FRET) acceptor of FAD fluorescence as illustrated by the overlap between the emission fluorescence spectrum of FAD and the absorbance spectrum (Figure 6A). Next, we calculated the distance for 50% FRET efficiency (R_0_) between FAD and Aβ(1–42) HiLyte^TM^-Fluor555 as described in Section 4, following a protocol used in previous works [61,62,63]. A value of 3.96 × 10^−3^ has been calculated for the quantum yield of FAD bound to the Cb5R (Φ_D_), using as reference the quantum yield of FMN given in standard fluorescence data tables. A value of the overlap integral J(λ) of 6.0254174 × 10^15^ cm^3^·M^−1^ has been calculated from the recorded emission spectrum of FAD bound to the Cb5R and the absorbance spectrum of Aβ(1–42) HiLyte^TM^-Fluor555. An R_0_ value of 2.77 nm has been obtained by applying the equation given in Section 4, assuming a random orientation between donor and acceptor.

Then, we recorded the emission spectrum of human recombinant Cb5R with a His_6_-tag in the absence and presence of 100 nM of Aβ(1–42) HiLyte^TM^-Fluor555 and the emission spectrum of 100 nM Aβ(1–42) HiLyte^TM^-Fluor555 in the absence of Cb5R (Figure 6B). Next, the latter spectrum, which is due to the direct excitation of the acceptor HiLyteFluor555, has been subtracted to the emission spectrum of Cb5R plus 100 nM of Aβ(1–42) HiLyte^TM^-Fluor555, and the result is shown in Figure 6C. We avoided the use of a saturating concentration of the Aβ(1–42)-induced increase in the FAD fluorescence in Cb5R shown in Figure 5 to prevent the complications of analysis coming from an excessively large peak of the HiLyte^TM^-Fluor555 fluorescence on the overall emission spectrum. The results shown in Figure 6C indicate that 100 nM of Aβ(1–42) HiLyte^TM^-Fluor555 produces a 45 ± 5% quenching of the peak donor FAD fluorescence at 525 nm, and a similar increase in the peak of the acceptor HiLyte^TM^-Fluor555 fluorescence at 555 nm. Thus, FRET measurements provide an additional proof of interaction between Cb5R with a His_6_-tag and Aβ(1–42) HiLyte^TM^-Fluor555.

## 3. Discussion

The results show the interaction between Aβ(1–42) HiLyte^TM^-Fluor555 and submicromolar concentrations of His_6_. Likely, this interaction will be further potentiated by transition metal ions, which bind with very high affinity to Aβ(1–42) [2,54], and, also, to His_6_ [64]. Indeed, we cannot completely exclude that trace amounts of transition metal ions, which are present in the preparations of commercial samples of Aβ peptides and in buffers, are playing a role in the Aβ(1–42) HiLyte^TM^-Fluor555/His6 complexes formed under our experimental conditions. The kinetics of the increase in Aβ(1–42) HiLyte^TM^-Fluor555 fluorescence intensity after the addition of His_6_ is a relatively slow process for this type of molecules, with a half-time of around 10 min, and an initial short lag phase within the first minute. Therefore, the kinetic results suggest the occurrence of conformational changes in the formation of a complex between Aβ(1–42) HiLyte^TM^-Fluor555 and His_6_. Also, it is to be noted that a concentration of 100 nM His_6_ is almost saturating for its complexation with 10 nM Aβ(1–42) HiLyte^TM^-Fluor555, indicating that the dissociation constant (Kd) of this complex is in the submicromolar concentration range. However, the maximum increase in the Aβ(1–42) HiLyte^TM^-Fluor555 fluorescence intensity (<10%) is not large enough over the noise of the data to obtain a reliable Kd value for this complex from the dependence upon the His_6_ concentration using this experimental approach.

Docking simulations provide a rational support for the formation of a complex between His_6_ and Aβ(1–42). The simulations performed using the CABS-dock Web Server, which do not require the input of an initial conformation of His_6_, yield three-dimensional structures for this complex that can be grouped in three-classes taking into account the Aβ(1–42) peptide domain directly interacting with His_6_. Thus, changes in the structure of His_6_ are one likely cause of the short initial lag phase of the kinetics of complex formation. On the other hand, the cluster of poses represented by cluster 1 of Figure 2, generated using docking simulations for the His_6_/Aβ(1–42) complex, predicts that His_6_ interacts with amino acid residues 24–42 of Aβ(1–42). In a previous work [40], we have shown that this domain of Aβ(1–42) is the most directly involved in the formation of Aβ(1–42)/CaM and Aβ(1–42)/calbindin-D28k complexes. However, docking analysis predicts a relevant contribution of LYS28 and LYS16 in the case of the cluster 1 of the His_6_/Aβ(1–42) complex, the first cluster ranked by energy calculations, while only highly hydrophobic amino acids of Aβ(1–42) are involved in the interaction interface with CaM and calbindin-D28k [40]. The contribution of electrostatic interactions and H-bonds in the interface of interaction between His_6_ and Aβ(1–42) is even stronger in the structures of the cluster 2 of His_6_/Aβ(1–42) complex, the second ranked by free energy calculations, as the buried surface area/ accessible solvent area ratio results in the following amino acid residues with higher participation in the interaction with the Aβ peptide: TYR10 > HIS6 > GLU3 > HIS14 > ASP7. Also, the presence of charged amino acids ASP1, GLU3, ARG5, ASP7 and GLU11 in the Aβ(1–42) domain of the interacting interface of cluster 3 of His_6_/Aβ(1–42) complex lends further support to the higher relevance of electrostatic interactions in the formation of this complex. Thus, docking analysis points out a higher contribution of electrostatic interactions and H-bonds in the His_6_/Aβ(1–42) complex than in Aβ(1–42)/CaM and Aβ(1–42)/calbindin-D28k complexes. Consistent with this analysis, in this work, we show that His_6_ efficiently antagonizes the interaction between Aβ(1–42) HiLyte^TM^-Fluor555 and these two proteins, with concentrations for 50% effect ≤1 μM of His_6_, values that are more than 10-fold higher than those obtained for the hydrophobic peptide VFAFAMAFML(amidated-C-terminus amino acid) in our previous work [40]. However, it is to be noted that the dye HiLyte^TM^-Fluor555 is covalently bound to the NH_2_-terminus amino acid of Aβ(1–42), which is distant from the amino acid residues 24–42 of Aβ(1–42). Since fluorescence measurements point out that the interaction of His_6_ with Aβ(1–42) HiLyte^TM^-Fluor555 alters the microenvironment of HiLyte^TM^-Fluor555, it is likely that the structure of the His_6_/Aβ(1–42) complex can be better described by a weighted combination of the three different clusters of His_6_/Aβ(1–42) complex shown in Figure 2 or that the molecular stoichiometry of this complex is higher than 1:1. Indeed, the cluster 2 in Figure 2, the second most probable model yielded by docking simulations, predicts the binding of His_6_ to the NH_2_-terminus domain of Aβ(1–42). Nevertheless, the possibility of a significant generation of 3His_6_/Aβ(1–42) complex is unlikely because of the large difference in free energies between clusters 1 and 3, and, also, because of steric hindrance between the His_6_ position in clusters 2 and 3. In addition, a shift between cluster structures during the formation of the His_6_/Aβ(1–42) complex could provide a simple explanation for the slow kinetics of interaction between His_6_ and Aβ(1–42) HiLyte^TM^-Fluor555. This is a plausible possibility because docking simulations do not yield large differences of the free energy changes predicted for the clusters 1 and 2 of the His_6_/Aβ(1–42) complex. Further extensive experimental studies will be required to obtain the molecular stoichiometry and structure of the His_6_/Aβ(1–42) complex with atomic resolution.

The high affinity of His_6_ for neurotoxic Aβ peptides, like Aβ(1–42) and Aβ(25–35), is further demonstrated by the results obtained in this work with human recombinant Cb5R with a His_6_-tag. First, the inhibition of the Cb_5_ reductase activity of human recombinant Cb5R with a His_6_-tag by Aβ(1–42) or Aβ(25–35) has a Ki of 50–60 nM of Aβ peptide monomers, while up to 2 μM of Aβ(1–42) monomers produce less than 10% inhibition of this activity of Cb5R after removal of the His_6_-tag. Second, this Ki value is not significantly different from the value of the apparent dissociation constant of the complex between Aβ(1–42) or Aβ(25–35) and human recombinant Cb5R with a His_6_-tag calculated from titrations with these Aβ peptides of the fluorescence of FAD, the prosthetic group of this enzyme. Since the predominant aggregation state of the Aβ(1–42) solutions used in our experimental conditions is the dimer, see Section 4, our results yield values ≤30 nM of Aβ(1–42) dimers for both Ki and K_d,app_. Thus, we conclude in this work that the His_6_-tag strongly potentiates the binding of the neurotoxic peptides Aβ(1–42) and Aβ(25–35) to the human recombinant Cb5R. In addition, our results point out that the Cb_5_ reductase activity of the human recombinant Cb5R minus the His_6_-tag is not significantly inhibited by up to 2 μM of Aβ(1–42) oligomers, an intracellular concentration that is unlikely to be reached within the cells because the critical concentration for the induction of Aβ(1–42) fibrillization is in the submicromolar range [34,35]. Of note, a ‘scrambled’ non-toxic Aβ(1–42) peptide used in previous works [27,30] does not bind to the His_6_-tag of the human recombinant Cb5R, nor produces a significant inhibition of its activity. As the insertion of a His_6_-tag is widely used to help with the purification of recombinant proteins, this conclusion strongly suggests removing it prior to using these recombinant proteins in studies dealing with the biochemical and biological effects of Aβ(1–42).

**Figure 7:**
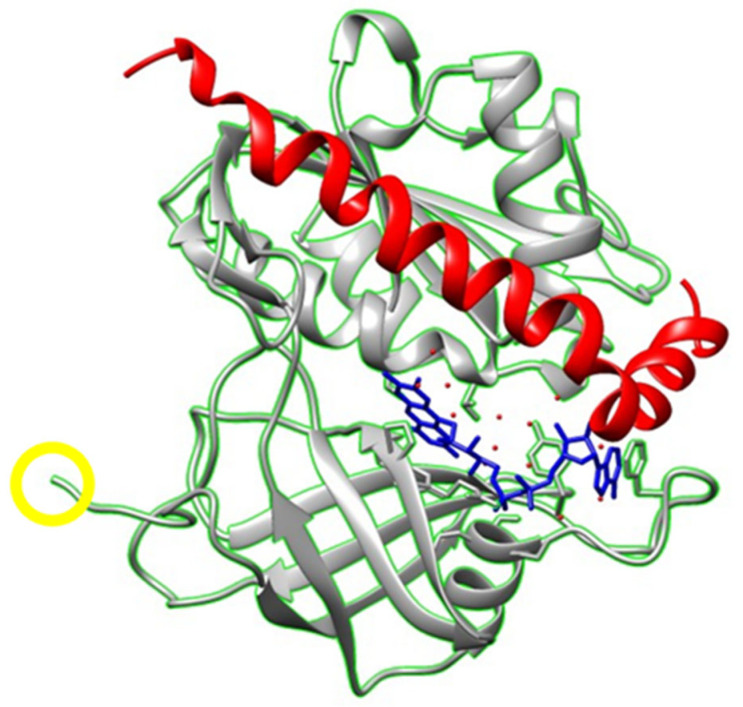
Simulation generated using docking between the soluble human erythrocytes isoform of Cb5R (PDB ID: 1UMK) and Aβ(1–42) (PDB ID: 1IYT) using the ClusPro Web Server. The polypeptide backbone of Cb5R is shown in gray/green, with the position of the prosthetic group FAD in blue, and the Aβ(1–42) is shown in red. The NH_2_-terminus amino acid of the Cb5R is labeled with a yellow circle.

The large increase in the intensity of the fluorescence of its prosthetic group FAD monitors the binding of Aβ(1–42) and Aβ(25–35) to human recombinant Cb5R with a His_6_-tag. Since the catalytic site does not overlap with the position of the His_6_-tag bound to the NH_2_-terminus amino acid of Cb5R, the inhibition by Aβ(1–42) or Aβ(25–35) of its Cb_5_ reductase activity is due to a long-distance induced conformational change. Taking into account that 100 nM of Aβ(1–42) HiLyte^TM^-Fluor555 lies between 60 and 65% of the saturating concentration of Cb5R (Figure 5), we can calculate that at a saturation by Aβ(1–42) HiLyte^TM^-Fluor555, the quenching of the donor FAD fluorescence will rise up to 70 ± 7%. As at this concentration of Aβ(1–42), the peptide will be in equilibrium between monomers and dimers [58], we have calculated the most probable distance range between FAD and the dye HiLyte^TM^-Fluor555 using the equation for FRET for 1 and 2 acceptors per donor with the assumption of random orientation given in Section 4. The upper distance limit of this range will be set by the case of the two acceptors HiLyte^TM^-Fluor555 dyes, one bound to each monomer, located equidistant from the donor FAD. The calculated distances between the donor FAD group of Cb5R and the acceptor HiLyte^TM^-Fluor555 dye covalently linked to Aβ(1–42) have been 2.35 nm and 3.1 nm for 1 or 2 equidistant acceptors, respectively. Of note, the measurements of the anisotropy of the FAD fluorescence, performed as indicated in Section 4, presented anisotropy values lower than 0.005. As noted in [65], with this low value of donor anisotropy, there is >90% probability that the above-calculated distance range is correct. In order to evaluate whether the structural coupling between the known structures of the Cb5R and Aβ(1–42) allows for the satisfaction of this requirement, we have performed docking between the soluble human erythrocytes isoform of Cb5R (PDB ID: 1UMK) and Aβ(1–42) (PDB ID: 1IYT) using the ClusPro Web Server as described in Section 4. Among the 10 model structures generated in silico, we selected the model structure of the complex between Cb5R and Aβ(1–42) shown in Figure 7, in which the Aβ(1–42) lies close to the His_6_-tag bound to the NH_2_-terminus amino acid of the human recombinant Cb5R. This model structure satisfies the above distance requirement between the NH_2_-terminus amino acid of the Aβ(1–42) and FAD derived from FRET measurements, which can be estimated between 2 and 3 nm by taking into account the uncertainty of the orientation and size of the HiLyte^TM^-Fluor555 dye in the complex. In addition, this model structure shows that the amino acids 25–35 of the Aβ(1–42) lies near the catalytic center, where the isoalloxazine ring of the FAD prosthetic group of the Cb5R is located. Also, the Cb_5_ binding site in the Cb5R, identified in previous works [66,67], lies close to the FAD group in the protein domain located as shown below in Figure 7. The extensive overlap of the 25–35 amino acid residues of the Aβ(1–42) with the Cb_5_ domain suggests that steric hindrance and/or an incorrect orientation of bound Cb_5_ could account for the inhibition of the Cb_5_ reductase activity measured in this work.

In summary, His_6_ binds with high affinity to Aβ(1–42), and at micromolar concentrations antagonizes the formation of Aβ(1–42)/CaM and Aβ(1–42)/calbindin-D28k complexes. In addition, a His_6_-tag provides a high-affinity site for the binding of neurotoxic Aβ(1–42) and Aβ(25–35) peptides to the human recombinant Cb5R, and sensitizes this enzyme to inhibition by these peptides. Therefore, our results suggest that a His_6_-tag could be used to experimentally direct the action of neurotoxic Aβ peptides toward selected cellular targets.

## 4. Materials and Methods

### 4.1. Chemicals

Human Aβ(1–42)-HiLyte^TM^-Fluor555 was purchased from AnaSpec (Freemont, CA, USA). Aβ(1–42), Aβ(25–35) and Aβ ‘scrambled’ were supplied by StabVida (Caparica, Portugal) and GenicBio Limited (Shanghai, China). His_6_ was purchased from Quimigen (Madrid, Spain). Bovine brain CaM and Thrombin Clean Cleavage^TM^ kit were purchased from Merck-Sigma-Aldrich (Madrid, Spain).

All the other chemicals used in this work were of analytical grade and supplied by Merck-Sigma-Aldrich (Madrid, Spain) and ThermoFisher Scientific (Madrid, Spain).

### 4.2. Preparation of Aβ(1–42) Solutions

Stock Aβ(1–42) solutions were prepared by dissolving the lyophilized peptide at 4 mg/mL in 1% NH_4_OH. Later, the stock Aβ(1–42) solution was diluted in phosphate-buffered saline to a concentration of 177 μM. The aggregation state of Aβ(1–42) in the 177 μM solution has been assessed in Tricine–sodium dodecyl sulfate–polyacrylamide gel electrophoresis (SDS-PAGE) as described in our previous works [27,40]. As shown in [27,40], in this solution, dimers account for approximately 90% of Aβ(1–42) and trimers for ≤10%, while monomers and higher molecular species are almost undetectable.

### 4.3. Preparation of the Human Recombinant Cb5R Soluble Isoform

Clones of the soluble isoform of human Cb5R prepared in a previous work were used to express and purify the recombinant protein purified as indicated in [67]. The purified protein was aliquoted and conserved in 30% glycerol at −80 °C until use.

**Figure 8:**
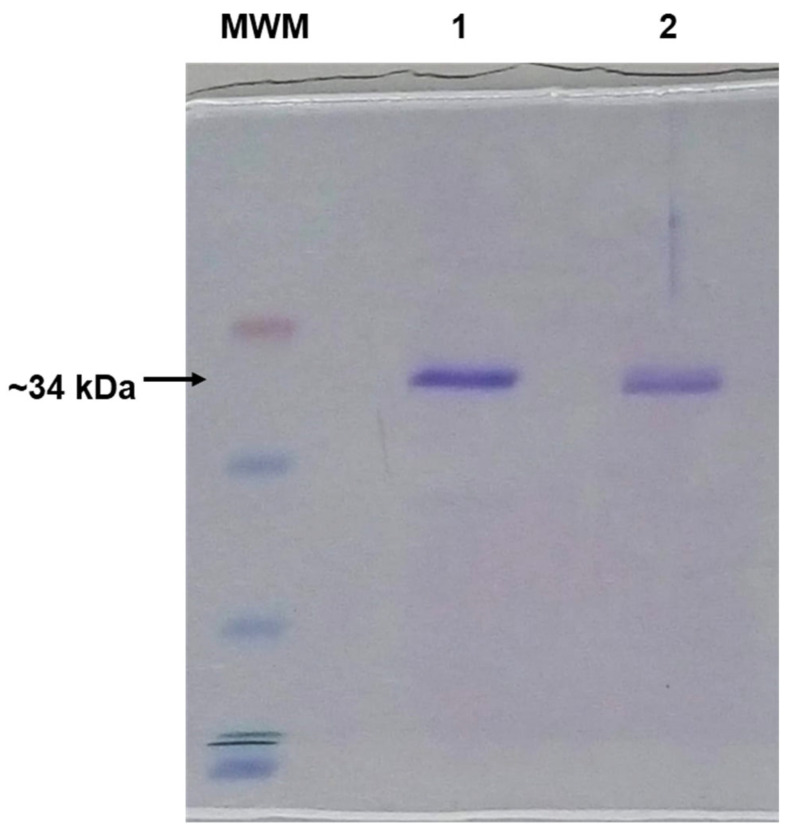
SDS-PAGE of human recombinant Cb5R. Lane 1: His_6_-tagged Cb5R. Lane 2: Cb5R after the removal of His_6_ by treatment with the Thrombin Clean Cleavage^TM^ kit and passage through a Sephadex G75 column. MWM, lane of molecular weight markers. The gel showed that the treatment with the Thrombin Clean Cleavage^TM^ kit produced the expected decrease of ~1 kDa in the molecular weight and no further proteolytic degradation of Cb5R.

The C-terminal His_6_-tag recombinant Cb5R has a thrombin-cutting site between the His_6_-tag and the enzyme, as previously shown [67]. The His_6_-tag was cut by overnight incubation at 4 °C with the Thrombin Clean Cleavage^TM^ kit. The recombinant Cb5R minus the His_6_-tag was separated from the cleaved peptide via column chromatography through Sephadex G75 (1 × 25 cm), and the efficient removal of the His_6_-tag was confirmed using SDS-PAGE (Figure 8).

The concentration of recombinant Cb5R was determined using the method of Bradford, with bovine serum albumin as standard or from absorbance measurements at 450 nm, using an extinction coefficient of 11.3 mM^−1^·cm^−1^ for the FAD prosthetic group [67,68].

### 4.4. Preparation of the Human Recombinant Calbindin-D28k and Cb_5_

The human recombinant calbindin-D28k and soluble isoform of Cb_5_ have been expressed and purified as described in detail in previous works [40,67,69]. The purified protein were aliquoted and conserved in 30% glycerol at −80 °C until use.

The concentration of recombinant Cb5R was determined using the method of Bradford with bovine serum albumin as standard or from absorbance measurements at 550 nm using a differential extinction coefficient between reduced and oxidized Cb_5_ of 16.5 mM^−1^·cm^−1^, as in previous works [67,69].

### 4.5. Measurements of His_6_, CaM and Calbindin-D28k Interaction with Aβ(1–42) HiLyte^TM^-Fluor555

The change of the fluorescence intensity of Aβ(1–42) HiLyte^TM^-Fluor555 has been used to monitor its complexation with His_6_, CaM and calbindin-D28k. Fluorescence measurements were performed using a Fluoromax+ fluorescence Spectrophotometer (Horiba Jobin Yvon IBH Ltd., Glasgow, UK) in quartz cuvettes of 1cm light pathlength with a total volume of 2.5 mL at room temperature (24–25 °C), with excitation and emission slits of 5 nm.

The measurements of the kinetics of fluorescence have been performed in buffer 50 mM *N*-[2-hydroxyethyl] piperazine-*N*′-[2-ethanesulfonic acid], 100 mM KCl and 50 µM CaCl_2_ (pH 7.05). The cuvette was kept with magnetic stirring in the dark within the cuvette holder of the fluorimeter until stabilization of the fluorescence intensity after the addition of 10 nM Aβ(1–42) HiLyte^TM^-Fluor555, routinely between 20 and 40 min. Then, His_6_ was added to the cuvette at the concentrations indicated in the figures, and the fluorescence intensity was recorded as a function of time with excitation and emission wavelengths of 520 nm and 567 nm, respectively. His_6_ was added from stock concentrated solutions prepared in the assay buffer, such that the total added volume was always lower than 10 μL. Control experiments were run by adding the same volume of the assay buffer, and showed no significant changes in the fluorescence intensity of Aβ(1–42) HiLyte^TM^-Fluor555.

In the experiments dealing with the effect of His_6_ on the kinetics of fluorescence intensity increase after the addition of 5 nM CaM or calbindin-D28k, these proteins were added after 15 min preincubation with the indicated concentration of His_6_. CaM and calbindin-D28k were added from concentrated stock solutions freshly prepared in the assay buffer used in the fluorescence measurements, such that the total added volume was always lower than 10 μL.

### 4.6. In Silico Docking between Aβ(1–42) and His_6_

These docking studies have been performed using the MDockPeP Server (https://zougrouptoolkit.missouri.edu/mdockpep/index.html), accessed on 26 November 2022. This server generates structural simulations of complexes between proteins and peptides, requiring only the input of the PDB file of the protein and the amino acids sequence of the peptide. In this work, we have used the Aβ(1–42) (PDB: 1IYT) as the protein partner. The server generates the 10 more probable poses for the complex formation, on the basis of the results after three major steps: (1) calculation of peptide conformers, in our case, of His_6_; (2) global flexible sampling of the binding modes protein–peptide; and (3) score and classification of the evaluated types of bonds involved in the formation of the complex [70]. Interacting interfaces were analyzed using PDBePISA [71], access date 4–5 October 2023, and they were visualized using UCSF Chimera [72].

### 4.7. Titration with Aβ(1–42) and Aβ(25–35) of the NADH-Dependent Cb_5_ Reductase Activity of the Cb5R

The measurements were performed with a Shimadzu UV1800 spectrophotometer at a wavelength of 550 nm in 1 mL cuvettes containing the assay buffer 20 mM phosphate/0.1 mM diethylenetriaminepentaacetic acid (pH 7), with 0.11 μg of Cb5R/mL and 5 μM of Cb_5_ in the absence and presence of the concentrations of Aβ(1–42), Aβ(25–35) and ‘scrambled’ Aβ(1–42), indicated in the figures. The reaction was started by the addition of 0.25 mM NADH after 10 min preincubation of the Cb5R in the assay medium, and initial rates were measured during periods of time ranging between 5 and 10 min, with a less than 10% drop in the Cb_5_ concentration in the assay cuvette. The Cb_5_ reductase activity was calculated from the change of absorbance at 550 nm using a differential extinction coefficient between reduced and oxidized Cb_5_ of 16.5 mM^−1^·cm^−1^, as in previous works [67,69]. The titration of the NADH-dependent Cb_5_ reductase activity of the Cb5R with the Aβ peptide was carried out by measuring in each assay cuvette the activity before and after the addition of each concentration of the Aβ peptide, in order to have an internal control of the activity in the absence of the Aβ peptide to cancel putative minor differences in Cb5R pipetting. The volume of Aβ peptide solution added ranged between 1 and 2.5 μL, pipetted from a freshly prepared solution of a prefixed concentration by dilution of aliquots of the stock solution in the assay buffer.

### 4.8. Titration with Aβ(1–42) and Aβ(25–35) of the FAD Fluorescence of Human Recombinant Cb5R with a His_6_-Tag and FRET Analysis

The FAD fluorescence of human recombinant Cb5R with a His_6_-tag (32 nM) has been measured at 25 °C with a Perkin Elmer 650-40 spectrofluorometer in quartz cuvettes of 1 cm pathlength with excitation and emission wavelengths of 460 nm and 520 nm, respectively, and excitation and emission slits of 10 nm. The assay buffer was 20 mM phosphate/0.1 mM diethylenetriaminepentaacetic acid (pH 7). We noticed that the FAD fluorescence of the recombinant enzyme displays a small but steady increase as a function of time. This indicates a weak instability of the recombinant Cb5R in these experimental conditions because its denaturation produces around a 1000-fold increase in the FAD fluorescence [67]. Due to this, the operational protocol for data acquisition was as follows: after recording the drift of the fluorescence intensity of the Cb5R in the assay buffer for 5 min, Aβ peptides were added at the concentrations indicated in the figures and the fluorescence intensity was recorded for another 5 min. The increase in fluorescence produced by each Aβ peptide concentration shown in the figures was corrected by the drift of fluorescence intensity drift recorded before the addition of the Aβ peptide. 

The steady-state anisotropy of fluorescence, r_s_, has been calculated from the polarization of fluorescence (P) using the equation [73]:

r_s_ = 2 × P/(3 − P). (1)

Polarization of fluorescence, P, was calculated using the equation:

P = [I_ǁ_ − G × I_Ʇ_]/[I_ǁ_ + G × I_Ʇ_], (2)

where I_ǁ_ and I_Ʇ_ are the fluorescence intensities measured with parallel (0°/0°) and perpendicularly (0°/90°) oriented polarizers, respectively, and G is the correction factor for polarization characteristics of the emission monochromator [62,63,73]. A value of G of 1.04 ± 0.02 has been obtained for our fluorimeter and used in our calculations.

The distance (d) between the Cb5R prosthetic group FAD and the dye HiLyte^TM^-Fluor555 covalently bound to Aβ(1–42), which form a donor–acceptor FRET pair, has been calculated for the cases of 1 and 2 acceptors per donor. In the case of multiple acceptors per donor, the rate constant of total FRET (k_T_) is the sum of the rate constant of FRET between each donor–acceptor pair (k_i_) that can be formed in the assembly [74,75,76,77]. The total FRET efficiency (E) is the sum of the FRET efficiency for each donor–acceptor pair that is possible with the geometrical constraints of the system [74,75,76]. For each donor–acceptor pair, we have used the equation for FRET: E = d^−6^/(R_0_^−6^ + d^−6^) [62]. In this equation, E is the FRET efficiency and R_0_ is the distance for 50% FRET efficiency between FAD and HiLyte^TM^-Fluor555. The value of R_0_ has been calculated in this work using the operational protocol followed from previous works for other FRET donor–acceptor pairs [61,62,63]. Briefly, we have applied the equation [62]:

R_0_ = 9.7 × 10^3^ × [K^2^ × ΦD × J(λ) × n^−4^]^1/6^ × Å (3)

with the assumption of random orientation between donor and acceptor (K^2^ = 2/3) and the relative refraction index of an aqueous medium (n = 1.33). The quantum yield of the donor (Φ_D_) and the overlap integral, J(λ), have been calculated as indicated in Section 2 of this article.

### 4.9. In Silico Docking Simulation between Aβ(1–42) and Cb5R

Docking between Aβ(1–42) and Cb5R has been performed using the ClusPro server with the following PDB files: Aβ(1–42) (PDB ID: 1IYT) and Cb5R (PDB ID: 1UMK), access date 29 January 2019. The images and analysis of the model structures were built up with the UCSF Chimera software.

### 4.10. Statistical Analysis

All the results are means ± standard error of the mean (S.E.M.) of experiments performed, at least, in triplicate.

## Figures and Tables

**Table 1:** Interacting interfaces for the complex formation between His_6_ and Aβ peptides obtained via molecular docking simulation.

Cluster	Involved Receptor Residues on the Interaction (Aβ Peptide)	Involved Ligand Residues on the Interaction (His_6_ Peptide)	Binding Energy Δ^i^G (kcal/mol)
Cluster 1	LYS16, LEU1, PHE20, LA21, VAL24, GLY25, LYS2, ILE31, LEU34, MET35,	HIS1, HIS3, HIS4, HIS5, HIS6	−6.8
Cluster 2	GLU3, HIS6, ASP7, TYR10, HIS14	HIS1, HIS2, HIS6	−4.8
Cluster 3	GLY9, VAL18	HIS1, HIS2, HIS6	−2.6

## Data Availability

Data supporting the reported results can be found in the laboratory archives of the authors.

## Supplementary Materials

The following supporting information can be downloaded at: https://www.mdpi.com/article/10.3390/molecules28207138/s1, Figure S1: PDB files of the ten most probable model structures generated using the MDockPeP Server for the His_6_/Aβ(1–42) complex; Table S1: Supplementary Table S1; Table S2: Supplementary Table S2; Table S3: Supplementary Table S3.

## Abbreviations

Aβ	amyloid β peptides
AD	Alzheimer’s disease
CaM	calmodulin
Cb_5_	cytochrome *b*_5_
Cb5R	cytochrome *b*_5_ reductase
ER	endoplasmic reticulum
FAD	flavine adenine dinucleotide
FMN	flavine mononucleotide
FRET	fluorescence resonance energy transfer
His_6_	hexa-histidine
K_d,app_	apparent dissociation constant
K_i_	inhibition constant
SDS-PAGE	sodium dodecyl sulfate polyacrylamide gel electrophoresis
S.E.M.	standard error of the mean
