# Supplementary material for: Hexa-Histidine, a Peptide with Versatile Applications in the Study of Amyloid-β(1–42) Molecular Mechanisms of Action
Source: Molecules. 2023 Oct 17;28(20):7138. doi: 10.3390/molecules28207138 (PMC10609148; doi:10.3390/molecules28207138)
Supplement: Supplementary file 1 [file molecules-28-07138-s001.zip › PDB files and SupplemTables/Supplementary Tables.pdf]

Supplementary tables with data obtained from models 1-10 clustering as cluster 1-3

Supplementary Table 1. Selected models of the His6: Aβ peptide complex forming the Cluster 1

Cluster 1	Receptor (Aβ peptide)	Ligand (His6)	H-bond	Salt bridges	Binding Energy ΔⁱG kcal/mol
Model 1	HIS13, **LYS16**, **LEU17**, **PHE20**, **ALA21**, **VAL24**, **GLY25**, **LYS28**, **ILE31**, **LEU34**, **MET35**, GLY38, VAL39, ALA42	**HIS1, HIS3, HIS4, HIS5, HIS6**	GLY38:HIS6<br>ALA42:HIS6	ALA42: HIS6	-6.6
Model 4	**LYS16**, **LEU17**, VAL18, **PHE20**, **ALA21**, GLU22, **VAL24**, **GLY25**, SER26, LYS28, **ILE31**, LEU34, **MET35**	**HIS1,** HIS2, **HIS3, HIS4, HIS5, HIS6**	LEU17:HIS4<br>ALA21:HIS3	GLU22: HIS1	-4.3
Model 7	**LYS16**, **LEU17**, VAL18, **PHE20**, **ALA21**, GLU22, ASP23, **VAL24**, **GLY25**, **LYS28**, ALA30, **ILE31**, GLY33, **LEU34**, **MET35**	**HIS1,** HIS2, **HIS3, HIS4, HIS5, HIS6**	**ALA21:HIS3**		**-6.8**
Model 8	HIS13, **LYS16**, **LEU17**, VAL18, **PHE20**, **ALA21**, **VAL24**, **GLY25**, LYS28, **ILE31**, **LEU34**, **MET35**, GLY38, VAL39, ALA42	**HIS1,** HIS2, **HIS3, HIS4, HIS5, HIS6**	GLY38:HIS3		-6.6
Selected amino acids constituting the interacting interface	**LYS16,LEU1,PHE20,LA21,VAL24,GLY25,LYS2,ILE31, LEU34,MET35,**	**HIS1,HIS3 HIS4,HIS5 ,HIS6**			**-6.8**

Supplementary Table 2. Selected models of the His6: Aβ peptide complex forming the Cluster 2

Cluster 2	Receptor (Aβ peptide)	Ligand (His6)	H-bond	Salt bridges	Binding Energy $\Delta^{i}G$ kcal/mol
Model 2	ASP1, ALA2, **GLU3**, **HIS6**, **ASP7**, **TYR10**, **HIS14**	**HIS1**, **HIS2**, HIS3, HIS4, HIS5, **HIS6**			-1.4
Model 5	ALA2, **GLU3**, **HIS6**, **ASP7**, **TYR10**, **HIS14**, LEU17	**HIS1**, **HIS2**, HIS3, HIS5, **HIS6**	HIS6: HIS5<br>HIS6:HIS6<br>GLY9:HIS1<br>GLY9:HIS1<br>TYR10:HIS1		-4.1
Model 9	**GLU3**, PHE4, **HIS6**, **ASP7**, **TYR10**, **HIS14**, GLN15, LEU17, VAL18	**HIS1**, **HIS2**, HIS5, **HIS6**	GLU3:HIS5<br>HIS6:HIS5<br>ASP7:HIS6	GLU3:HIS6<br>GLU11:HIS2	**-4.8**
Model 10	ALA2, **GLU3**, **HIS6**, **ASP7**, **TYR10**, **HIS14**	**HIS1**, **HIS2**, HIS3, HIS4, **HIS6**	ALA2:HIS3<br>GLU3:HIS2<br>GLU3:HIS1<br>ASP7:HIS2<br>GLY9:HIS6	GLU3:HIS1<br>ASP7:HIS2	-3.3
Selected amino acids constituting the interacting interface	**GLU3**, **HIS6**, **ASP7**, **TYR10**, **HIS14**	**HIS1**, **HIS2**, **HIS6**			**-4.8**

Supplementary Table 3. Selected models of the His6: Aβ peptide complex forming the Cluster 3

Cluster 3	Receptor (Aβ peptide)	Ligand (His6)			Binding Energy $\Delta^{i}G$ kcal/mol
Model 3	**GLY9**, TYR10, VAL12, HIS13, HIS14, LEU17, **VAL18**	HIS1, **HIS2**, HIS3, HIS4, HIS5, HIS6	TYR10: HIS6<br>HIS14:HIS4		-2.6
Model 6	ASP7, SER8, **GLY9**, GLU11, **VAL12**, GLN15, LYS16, PHE19	HIS1, **HIS2**, HIS6	GLN15:HIS3<br>ASP7:HIS2<br>GLN15:HIS2	ASP7:HIS2<br>ASP7:HIS2<br>GLU11:HIS2<br>GLU11:HIS2	-1.7
Selected amino acids constituting the interacting interface	**GLY9**, **VAL18**	**HIS1**, **HIS2**, **HIS6**			**-2.6**
